# Exosomes Regulate Interclonal Communication on Osteogenic Differentiation Among Heterogeneous Osteogenic Single-Cell Clones Through PINK1/Parkin-Mediated Mitophagy

**DOI:** 10.3389/fcell.2021.687258

**Published:** 2021-09-17

**Authors:** Dongdong Fei, Yanmin Xia, Qiming Zhai, Yazheng Wang, Feng Zhou, Wanmin Zhao, Xiaoning He, Qintao Wang, Yan Jin, Bei Li

**Affiliations:** ^1^State Key Laboratory of Military Stomatology, National Clinical Research Center for Oral Diseases, Shaanxi International Joint Research Center for Oral Diseases, Center for Tissue Engineering, School of Stomatology, The Fourth Military Medical University, Xi’an, China; ^2^State Key Laboratory of Military Stomatology, National Clinical Research Center for Oral Diseases, Shaanxi Engineering Research Center for Dental Materials and Advanced Manufacture, Department of Periodontology, School of Stomatology, The Fourth Military Medical University, Xi’an, China; ^3^State Key Laboratory of Military Stomatology, National Clinical Research Center for Oral Diseases, Shaanxi Clinical Research Center for Oral Diseases, Department of Orthodontics, School of Stomatology, The Fourth Military Medical University, Xi’an, China

**Keywords:** mesenchymal stem cells, cell differentiation, cell communication, exosomes, osteogenesis, mitophagy

## Abstract

Mesenchymal stem cells (MSCs) are intrinsically heterogeneous and are comprised of distinct subpopulations that differ in their differentiation potential. A deeper understanding of the heterogeneity and intercellular communication within these heterogeneous subpopulations has significant implications for the potential of MSC-based therapy from the bench to the clinic. Here, we focused on the clonal osteogenic heterogeneity of periodontal ligament stem cells (PDLSCs) and explored how interclonal communication affects the osteogenic differentiation among these heterogeneous single-cell colonies (SCCs), and sought to determine the underlying mechanisms. Alkaline phosphatase (ALP) and Alizarin red staining identified the presence of SCCs with high (H-SCCs) and low osteogenic ability (L-SCCs). Conditioned medium derived from H-SCCs (H-CM) promoted mineralized nodule formation to a greater extent than that derived from L-SCCs (L-CM), which served as the target cells (TCs). However, treatment with the exosome biogenesis/release inhibitor GW4869 reduced the H-CM- and L-CM-related osteogenic differentiation-promoting potential. We further found that exosomes secreted by H-SCCs (H-Exo) were superior to those secreted by L-SCCs (L-Exo) in promoting the osteogenic differentiation of TCs. Mechanistically, TCs stimulated with H-CM and H-Exo exhibited higher levels of PINK1/Parkin-mediated mitophagy, while gain- and loss-of-function experiments showed that PINK1/Parkin-mediated mitophagy was positively associated with SCC osteogenic differentiation. Furthermore, PINK1 knock-down in H-Exo- and L-Exo-stimulated TCs inhibited their osteogenic differentiation through inhibiting PINK1/Parkin-mediated mitophagy. Our study uncovers a previously unrecognized mechanism that an exosome-mediated PINK1/Parkin-dependent mitophagy regulates interclonal communication among SCCs with osteogenic heterogeneity.

## Introduction

Periodontal ligament stem cells (PDLSCs), a type of mesenchymal stem cell (MSC), play an important role in the restoration and regeneration of alveolar bone (Huang et al., 2009). Although these cells have been used to treat alveolar bone loss in periodontitis patients, their therapeutic effect remains controversial (Chen et al., 2016). MSCs are intrinsically heterogeneous and are comprised of multiple distinct subpopulations (Graf and Stadtfeld, 2008; Nombela-Arrieta et al., 2011). Accordingly, knowledge regarding the heterogeneity and intercellular communication within these subpopulations has significant implications for the potential of MSC-based therapy from the bench to the clinic (Mendicino et al., 2014; Lech et al., 2016). Studies have indicated that PDLSC-derived single-cell colonies (SCCs) are heterogeneous in their osteogenic differentiation potential (Saito et al., 2014); however, little is known about how these SCCs coordinate and influence their osteogenic differentiation, which greatly limits the efficacy of a PDLSC-based therapy for bone regeneration.

The heterogeneity of mitochondrial function among different clones is closely related to cell function (Rossi et al., 2010; Ramirez et al., 2013). PINK1/Parkin-dependent mitophagy, a process that leads to the selective degradation of damaged mitochondria, is critical for mitochondrial quality control. Under certain insults, PINK1, an integral mitochondrial membrane protein, accumulates at the mitochondrial outer membrane, leading to the recruitment of the E3 ligase Parkin, and the consequent degradation of the damaged mitochondria in a proteasome-dependent manner (Kawajiri et al., 2011). Several recent studies have provided novel insights into the regulation of PINK1/Parkin-dependent mitophagy on the osteogenic differentiation of MSCs (Marycz et al., 2016; Pei et al., 2018). PINK1/Parkin-mediated mitophagy has been reported to regulate the osteogenic differentiation of dental pulp stem cells through the BMP/Smad pathway (Pei et al., 2018). Nevertheless, no study has shed light on the role of PINK1/Parkin-mediated mitophagy in interclonal osteogenic heterogeneity and osteogenic differentiation-related communication.

Exosomes, as vehicles for the transfer of mRNAs, miRNAs, and proteins, among other factors, are integral to intercellular communication (Tkach and Thery, 2016; He et al., 2018) and have key roles in a variety of cell functions, including proliferation (Okoye et al., 2014), differentiation (Kilchert et al., 2016), and apoptosis (Rivoltini et al., 2016). A recent study showed that exosomes from brown adipose tissue could significantly improve the oxygen consumption in hepatocytes through the regulation of the mitochondrial function (Zhou et al., 2020), suggesting that heterogeneous osteogenic SCCs may regulate osteogenic differentiation through an exosome-mediated regulation of the mitochondrial function.

In this study, we found that SCCs with a high osteogenic ability (H-SCCs) had a greater capacity than those with a low osteogenic ability (L-SCCs) to promote osteogenic differentiation, an effect that was mediated through exosomes. Mechanistically, we show that PINK1/Parkin-mediated mitophagy regulated osteogenesis among SCCs both *in vitro* and *in vivo*, while H-SCCs promoted the osteogenic differentiation of L-SCCs, which served as target cells (TCs), through the exosome-mediated activation of PINK1/Parkin-dependent mitophagy. This study uncovered a previously unrecognized relationship between PINK1/Parkin-mediated mitophagy and osteogenic heterogeneity among different SCCs and showed for the first time that an exosome-mediated PINK1/Parkin-dependent mitophagy regulates interclonal communication among SCCs with osteogenic heterogeneity. Importantly, our study will contribute to improving the efficacy of MSC-based therapy for the treatment of a variety of diseases.

## Materials and Methods

### Cell Culture

Samples of teeth extracted from healthy individuals for orthodontic purposes were obtained from the Department of Oral and Maxillofacial Surgery, School of Stomatology, The Fourth Military Medical University. All donors (aged 20–30 years) provided a written informed consent. PDLSCs were isolated and cultured as previously described (Liu et al., 2011; Zhang et al., 2012). Briefly, the periodontal ligament was separated from the middle third of the root surface, digested with 3 mg/ml collagenase I (Sigma-Aldrich Corp., St Louis, MO, United States) for 1 h at 37°C, and then cultured in α-MEM (Invitrogen, Carlsbad, CA, United States) supplemented with 10% fetal bovine serum (FBS) (Thermo Electron, Melbourne, VIC, Australia), 0.292 mg/ml glutamine (Invitrogen), 100 U/ml penicillin, and 100 mg/ml streptomycin. At 30% confluence, PDLSCs were digested and SCCs were obtained by limiting dilution. Briefly, the PDLSC suspension was serially diluted to 10 cells/ml, and then 0.1 ml of the suspension was seeded into each well of a 96-well culture dish. The next day, the number of cells in each well was determined and the wells containing only one cell were selected. After 3–4 weeks of culture, these SCCs were digested and seeded in 6-well culture dishes. Finally, approximately 15–30 clones from one individual were selected. For the study, SCCs at passages 4 to 5 were used, with the medium being changed every 3 days. For cell sheet formation, 1 × 10^6^ cells were seeded in 6-well dishes with a medium containing 50 μg/ml vitamin C and cultured for 14 days.

### Flow Cytometric Analysis

A total of 2 × 10^5^ SCCs were incubated at 4°C away from light for 1 h with antibodies targeting the FITC isotype control (400107), PE isotype control (400112), CD34 (343506), CD45 (304008), CD73 (344015), CD105 (800504) (all from BioLegend, San Diego, CA, United States), and CD90 (12-0909-41, eBioscience, San Diego, CA, United States). After washing twice with PBS, the cells were subjected to a flow cytometric analysis (Beckman Coulter, Fullerton, CA, United States).

### Osteogenic Differentiation

Before the induction of osteogenesis, 2 × 10^5^ cells were seeded per well of 12-well culture dishes (Corning, New York, NY, United States) until 80–90% confluence, following which, the medium was changed to an osteogenic-inducing fluid (100 μg/ml ascorbic acid, 2 mol/L β-glycerophosphate, and 10 nmol/L dexamethasone; Sigma-Aldrich Corp.). After 7 days of culture, the expression levels of the osteogenesis-related genes were assessed by reverse transcription-quantitative PCR (RT-qPCR). Simultaneously, alkaline phosphatase (ALP) staining was also performed using a BCIP/NBT alkaline phosphatase color development kit (Beyotime Institute of Biotechnology, Shanghai, China). Alizarin red staining was used to assess the mineralized nodule formation 21–28 days after the induction of osteogenesis. For Alizarin red staining quantification, absorbance was measured at 562 nm after the addition of 10% cetylpyridinium chloride.

### Adipogenic Differentiation

A total of 2 × 10^5^ SCCs were seeded per well of 12-well culture dishes until 80–90% confluence. The culture medium was then replaced with adipogenic induction medium (0.5 μM dexamethasone, 0.5 mM isobutyl methylxanthine, 60 μM indomethacin, and 10 μg/ml insulin; Sigma-Aldrich Corp.) and the cells were stained with Oil Red O on day 21.

### Transfection Assays

For overexpression experiments, the PINK1 cDNA was amplified using the primer pair 5′-CGCAAATGGGCGGTAGGCGTG-3′ (forward) and 5′-TAGAAGGCACAGTCGAGG-3′ (reverse). Before transfection, SCCs were seeded in 12-well culture dishes and grown to 80–90% confluence, and then serum-starved for 2 h. The overexpression plasmid [pcDNA3.1(+)-PINK1-V5] or the empty vector [pcDNA3.1(+)-MCS-V5] (both from OBiO, Shanghai, China) was then transfected into the SCCs using Lipo2000 (Invitrogen). For siRNA experiments, SCCs were transfected with 50 nM PINK1 siRNA (sc-44598) or scrambled siRNA (sc-37007) (both from Santa Cruz Biotechnology, Santa Cruz, CA, United States).

After transfection, the SCCs were collected at 48 h for RNA detection and 72 h for protein detection. For osteogenic induction, the transfection medium was replaced with an osteogenic induction medium the following day.

### Conditioned Medium Treatment

Single-cell colonies with low and high osteogenic ability were seeded in 100-mm culture dishes until 80–90% confluence. Medium collected after 24 h of culture was used to stimulate the TCs. After the conditioned medium treatment for 48 h, the medium was replaced by the collected medium from L-SCCs and H-SCCs. For osteogenic induction, the medium was collected from L-SCCs and H-SCCs after 24 h of culture and mixed with equal volumes of twofold concentrated osteogenic induction medium, and then applied to the TCs for 48 h, followed with the replacement by a newly mixed medium. This procedure was repeated throughout the entire conditioned medium treatment experiment, which means that the mixed conditioned medium was maintained for the duration of both RT-qPCR and Alizarin red analysis.

### Reverse Transcription-Quantitative PCR Analysis

RNA was extracted using TRIzol reagent (Invitrogen) according to the protocol provided by the manufacturer. Reverse transcription and qPCR were performed using the PrimeScript RT Reagent Kit and the SYBR Premix Ex Taq kit (Takara, Tokyo, Japan), respectively. Target gene expression was normalized to that of *GAPDH*, and the 2^–ΔΔCt^ method was used. The primers used for qPCR include the following sequences: *RUNX2* forward, 5′-CGGAATGCCTCTGCTGTTATG-3′; *RUNX2* reverse, 5′-AAGG TGAAACTCTTGCCTCGTC-3′; *OCN* forward, 5′-GCCACCGA GACACCATGAGA-3′; *OCN* reverse, 5′-AGGCTGCACCTTTG CTGGAC-3′; *GAPDH* forward, 5′-GCACCGTCAAGGCTGAG AAC-3′; *GAPDH* reverse, 5′-TGGTGAAGACGCCAGTGGA-3′.

### Western Blot

Cells were washed twice with PBS and then lysed in the RIPA lysis buffer (Sigma-Aldrich Corp.) at 4°C. After centrifugation, soluble proteins were quantified by BCA assay (Beyotime). Equal amounts of protein were separated by 15 Tris-glycine SDS–polyacrylamide gel (Invitrogen) electrophoresis, transferred to a PVDF membrane (Merck Millipore, Billerica, MA, United States), blocked with 5% BSA in PBST (PBS with 0.1% Tween), and incubated overnight at 4°C with primary antibodies targeting GAPDH (CW0100, CWBIO, Beijing, China), CD9 (ab92726, Abcam, Cambridge, United Kingdom), TSG101 (ab125011, Abcam), CD81 (sc-9158, Santa Cruz Biotechnology, Texas, TX, United States), PINK1 (#6946), Parkin (#4211), and LC3-I/II (#12741) (all from Cell Signaling Technology, Danvers, MA, United States). After incubation with the respective secondary antibodies (Jackson ImmunoResearch Laboratories, West Grove, PA, United States), the bands were visualized using enhanced chemiluminescence (Pierce, Rockford, IL, United States) and quantified using ImageJ (Media Cybernetics, Silver Springs, MD, United States).

### Exosome Isolation and Identification

The culture medium was replaced with a serum-free culture medium when SCCs had reached 80–90% confluency. Exosomes were isolated from cell culture supernatants from L-SCCs and H-SCCs after 24–48 h of culture by differential ultracentrifugation. Briefly, the cell culture medium was centrifuged at 300 × *g* for 10 min, the supernatant was collected, centrifuged at 2,000 × *g* for 10 min, the supernatant collected again, and centrifuged at 16,000 × *g* for 30 min. The resulting supernatant went through two rounds of centrifugation at 100,000 × *g* for 70 min. The exosome-containing pellet was resuspended in PBS for the identification of functional assays. For identification, the morphology and size of the exosomes were assessed by the transmission electron microscopy (TEM) (HITACHI, H-7650, Tokyo, Japan) and ZetaView analyzer (Particle Metrix, Pmx110, Meerbusch, Germany). Exosomes were quantified using a BCA assay and 20 μg/ml exosomes were used for the functional assays. For osteogenic differentiation assay, osteogenic inducing fluid containing 20 μg/ml exosomes were used for the duration of both RT-qPCR and Alizarin red, with the medium being changed every 3 days.

### Exosome Inhibition Assay

GW4869 (HY-19363, MedChem Express, Monmouth Junction, NJ, United States), an inhibitor of exosome biogenesis and release, was dissolved in dimethyl sulfoxide (DMSO) (MP Biomedicals, Irvine, CA, United States) and added to L-SCCs and H-SCCs at a final concentration of 5 μM for 48 h. After three PBS washes, L-SCCs and H-SCCs were cultured in α-MEM for 24 h, and the collected supernatants were mixed with equal volumes of twofold concentrated osteogenic induction medium for TC stimulation. TCs were stimulated by the mixed medium for the duration of both RT-qPCR and Alizarin red analysis.

### Fluorescence Microscopy

LysoTracker Green (Yeasen Biotech Co., Ltd., Shanghai, China) and MitoTracker Deep Red (Thermo Fisher Scientific, Waltham, MA, United States) double staining was used for the evaluation of mitophagy. Briefly, SCCs were incubated in α-MEM supplemented with 50 nM LysoTracker Green and 100 nM MitoTracker Deep Red at 37°C for 40 min. After two washes, the medium was replaced with fresh α-MEM, and images of live cells were captured using a confocal microscope (Olympus, Tokyo, Japan). Colocalization was quantified using ImageJ.

### Animal Experiments

All animal experimental protocols in this study were authorized by the Animal Care Committee of the Fourth Military Medical University (2018-kq-059) and were performed according to the guidelines stipulated by the Animal Care Committee. Eight-week-old male Sprague-Dawley (SD) rats weighing 280–300 g were used. A total of 21 rats were randomly distributed into three groups, seven rats per group. The investigators were not blinded to the group allocation and the sample size was not predetermined by statistical analysis. The surgical procedure was performed as previously described (Sun et al., 2017). Briefly, after anesthetizing the rats and removing the hair on one cheek, an incision was made parallel to the line connecting the inner and outer canthi (from the corner of the mouth to the lower edge of the mandible) to expose the masseter muscle. After cutting off the masseter muscle and periosteum, a dental handpiece (SolelyBio, Shanghai, China) was used to create a periodontal defect located approximately 1.5 mm from the alveolar ridge crest of the first and second molars. Once the 2-mm long, 1.5-mm wide, and 1-mm thick defects had been established, they were filled in with cell sheets derived from empty vector-overexpressing L-SCCs, empty vector-overexpressing H-SCCs, or PINK1-overexpressing L-SCCs. After 4 weeks, the rats were euthanized and samples were fixed for a Micro-CT analysis of bone mineral density (BMD), bone volume/total volume (BV/TV), trabecular number (Tb.N), trabecular thickness (Tb.Th), and trabecular separation (Tb.Sp). The area for Micro-CT analysis was a cuboid (2-mm long, 1.5-mm wide, and 1-mm thick).

### Hematoxylin and Eosin Staining

Harvested mandibles were decalcified in 10% ethylenediaminetetraacetic acid (EDTA) for 6 weeks and then sectioned parallel to the long axis of the tooth. The bone defects were located through the histologically visible edges, and the samples were stained with hematoxylin and eosin (H&E) and observed under a microscope (Leica DM6 B, Wetzlar, Germany).

### Statistical Analysis

GraphPad Prism 8.0 was used for statistical analysis. All the experiments were repeated three times and the data are presented as means ± SD. The Student’s *t*-test was used for comparisons between two groups, and one-way ANOVA was used for comparisons among multiple groups. For western blot analysis, one tailed ratio paired *t*-test was performed. A *P*-value < 0.05 was considered statistically significant.

## Results

### Single-Cell Colonies With High Osteogenic Ability Show Greater Mineralization Promotion Ability Than Single-Cell Colonies With Low Osteogenic Ability

**FIGURE 1:**
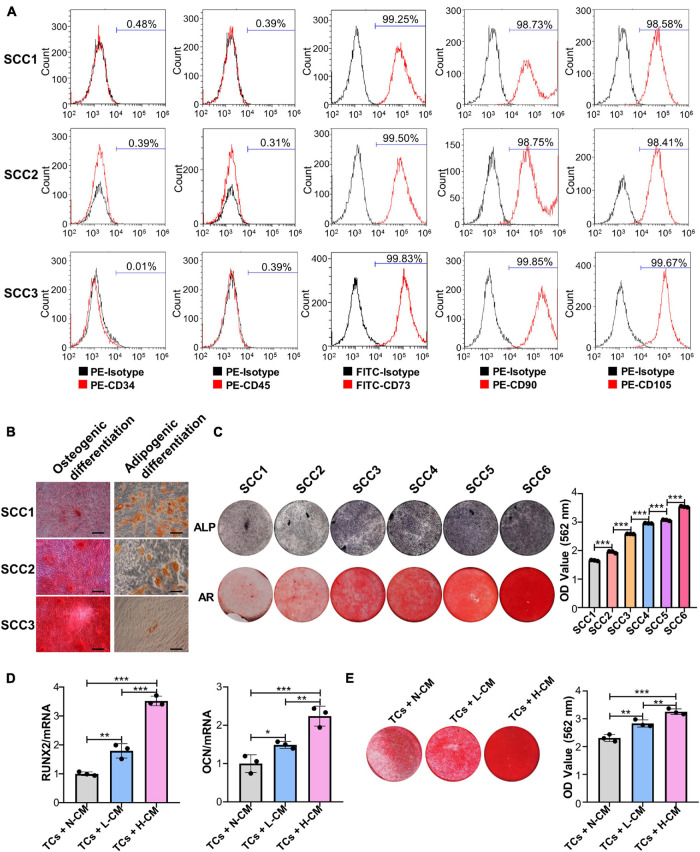
Single-cell colonies with high osteogenic ability had a greater mineralization promotion ability than L-SCCs. **(A)** The presence of cell surface markers of three SCCs was detected by flow cytometry. **(B)** The osteogenic and adipogenic differentiation potential of the three SCCs were assessed by Alizarin red and Oil Red O staining. **(C)** The osteogenic differentiation abilities of SCCs were tested by alkaline phosphatase (ALP) and Alizarin red staining. Alizarin red staining was quantified after the addition of 10% cetylpyridinium chloride. **(D)** Conditioned medium collected from H-SCCs or L-SCCs was mixed with equal volumes of twofold concentrated osteogenic induction medium, and was used to stimulate the TCs toward osteogenic differentiation for 7 days, following which, the levels of the osteogenic differentiation-related genes in the TCs were tested by RT-qPCR (*n* = 3). **(E)** Conditioned medium collected from H-SCCs or L-SCCs was mixed with equal volumes of twofold concentrated osteogenic induction medium, and was used to stimulate the TCs toward osteogenic differentiation for 28 days, following which, the TCs were subjected to Alizarin red staining (*n* = 3). CM, conditioned medium; TCs, target cells; AR, Alizarin red; N-CM, normal culture medium; L-CM, conditioned medium from L-SCCs; H-CM, conditioned medium from H-SCCs. Scale bar, 50 μm. *P < 0.05; **P < 0.01; ***P < 0.001.

The results of the flow cytometry assay showed that three randomly chosen SCCs derived from the same patient were positive for CD73, CD90, and CD105 and negative for CD34 and CD45 (Figure 1A). Alizarin red and Oil Red O staining analysis showed that these clones could be induced to differentiate into osteoblasts and adipocytes (Figure 1B). These results indicated that the PDLSC-derived SCCs had MSC characteristics. ALP and Alizarin red staining further showed that clear differences in the osteogenic potential existed between these clones. Some had a low osteogenic ability, as evidenced by the absence of mineralization nodules when observed under a microscope, while a few had a very high osteogenic ability (Figure 1C). SCCs displaying OD values not less than 2.5 for Alizarin red staining were defined as H-SCCs, while those with OD values smaller than 2.5 were defined as L-SCCs. Next, we used L-SCCs as the TCs to explore the effects of intercellular communication on the osteogenic differentiation potential of SCCs. Conditioned medium collected from H-SCCs (H-CM) or L-SCCs (L-CM) was used to stimulate the TCs toward osteogenic differentiation for 7 days. QPCR analysis showed that both L-CM and the H-CM could promote the expression of the osteogenesis-related genes runt-related transcription factor 2 (*RUNX2*) and osteocalcin (*OCN*), although the effect was stronger with H-CM treatment (Figure 1D). Alizarin red staining analysis also showed that both L-CM and H-CM could promote the formation of mineralization nodules in the TCs after 28 days of osteogenic induction, although a greater number of nodules was seen with H-CM stimulation (Figure 1E). We also investigated the effect of L-CM on the osteogenic differentiation of H-SCCs, with RT-qPCR and Alizarin red staining analysis indicating that L-CM treatment did not significantly promote the expression of the osteogenic-related genes *RUNX2* and *OCN* in H-SCCs (Supplementary Figure 1A) and increase the formation of mineralized nodules in H-SCCs (Supplementary Figure 1B).

### Single-Cell Colonies With High Osteogenic Ability Promotes the Osteogenic Differentiation of Target Cells Through Exosomes

**FIGURE 2:**
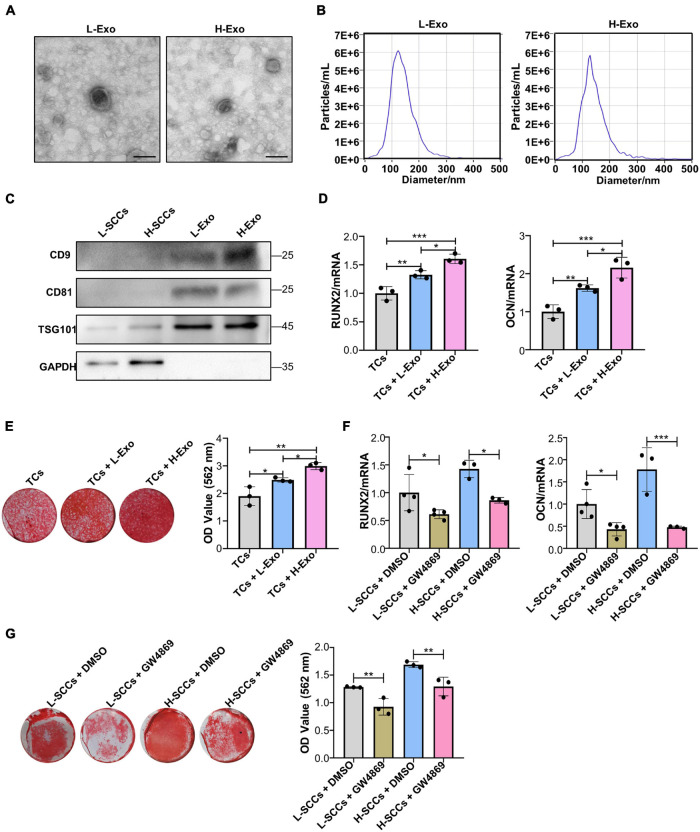
H-Exo were superior to L-Exo in promoting osteogenic differentiation in the TCs. **(A)** Exosomes obtained from H-SCCs and L-SCCs were identified by TEM. **(B)** Size distribution was assessed using a ZetaView nanoparticle tracking analyzer. **(C)** The expression of the exosomal marker proteins CD9, CD81, and TSG101 was tested by western blot. **(D)** Osteogenic inducing fluid containing 20 μg/ml exosomes was used, and the expression of the osteogenic differentiation-related genes in unstimulated TCs, or TCs stimulated by L-Exo or H-Exo, was measured by RT-qPCR after 7 days of osteogenic induction (*n* = 3). **(E)** Osteogenic inducing fluid containing 20 μg/ml exosomes was used, and Alizarin red staining of unstimulated TCs, or TCs stimulated by L-Exo or H-Exo, was performed after 28 days of osteogenic induction (*n* = 3). **(F)** The collected supernatants from the culture of DMSO- or GW4869-treated SCCs were mixed with equal volumes of twofold concentrated osteogenic induction medium to stimulate the TCs, and the expression levels of the osteogenic differentiation-related genes in the TCs were determined by RT-qPCR after 7 days of osteogenic induction (*n* = 3 or 4). **(G)** The collected supernatants from the culture of DMSO- or GW4869-treated SCCs were mixed with equal volumes of twofold concentrated osteogenic induction medium to stimulate the TCs, and Alizarin red staining of the TCs was performed after 28 days of osteogenic induction (*n* = 3). L-Exo, exosomes secreted by L-SCCs; L-SCCs + DMSO, TCs stimulated by the culture of DMSO-treated L-SCCs; L-SCCs + GW4869, TCs stimulated by the culture of GW4869-treated L-SCCs; H-Exo, exosomes secreted by H-SCCs; H-SCCs + DMSO, TCs stimulated by the culture of DMSO-treated H-SCCs; H-SCCs + GW4869, TCs stimulated by the culture of GW4869-treated H-SCCs; TCs, target cells; TEM, transmission electron microscopy. Scale bar, 100 nm. *P < 0.05; **P < 0.01; ***P < 0.001.

Differential ultracentrifugation was used to extract the exosomes secreted by H-SCCs (H-Exo) and L-SCCs (L-Exo) and the substances obtained were identified by TEM, ZetaView analysis, and western blot. The TEM results showed that the extracted substances displayed a double-layered membrane structure (Figure 2A). Nanoparticle tracking analysis with ZetaView showed that the diameters of the substances ranged from 100 to 150 nm (Figure 2B), which was consistent with previous studies reporting that exosome diameters range between 30 and 150 nm. Western blot analysis showed that these substances expressed the exosomal marker proteins CD9, CD81, and TSG101 (Figure 2C). These results indicated that the extracted substances were indeed exosomes. Subsequently, H-Exo and L-Exo (final concentration: 20 μg/ml) were used to stimulate the TCs under osteogenic induction conditions. RT-qPCR analysis showed that the expression of *RUNX2* and *OCN* was higher in the exosome-stimulated TCs than in the non-stimulated TCs (Figure 2D), while Alizarin red staining analysis also showed that the exosome-stimulated TCs had a greater osteogenic differentiation capacity (Figure 2E). Furthermore, RT-qPCR and Alizarin red staining results both demonstrated that H-Exo were superior to L-Exo in promoting the osteogenic differentiation of TCs. We also investigated the effect of L-Exo on the osteogenic differentiation of H-SCCs, and RT-qPCR and Alizarin red staining analysis indicating that L-Exo treatment had, to a certain extent, promoted the expression of the osteogenic-related gene *OCN* in H-SCCs (Supplementary Figure 2A). However, the expression of *RUNX2* and the formation of mineralized nodules in H-SCCs were not significantly changed after L-Exo treatment (Supplementary Figure 2B). We also performed an exosome inhibition assay using the exosome biogenesis/release inhibitor GW4869 to treat L-SCCs or H-SCCs. RT-qPCR and Alizarin red staining analysis both showed that GW4869 treatment significantly suppressed L-SCCs or H-SCCs-related osteogenic differentiation-promoting potential, as evidenced by the reduced expression of *RUNX2* and *OCN* and lower number of mineralized nodules in TCs from the GW4869-treated groups (Figures 2F,G).

### Single-Cell Colonies With High Osteogenic Ability Activates PINK1/Parkin-Mediated Mitophagy in Target Cells Through Exosomes

**FIGURE 3:**
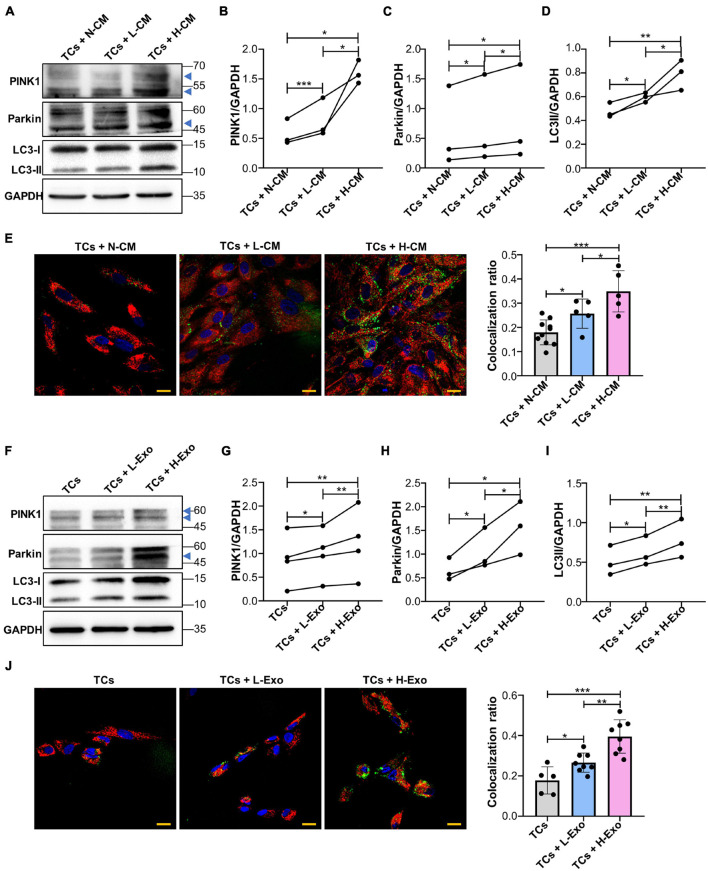
Single-cell colonies with high osteogenic ability activated PINK1/Parkin-mediated mitophagy in TCs through exosomes. **(A–D)** Proteins associated with PINK1/Parkin-mediated mitophagy in the TCs stimulated with N-CM, L-CM, and H-CM were tested by western blot and quantified using ImageJ (*n* = 3). **(E)** The colocalization of mitochondria and lysosomes in the TCs was assessed by fluorescence microscopy after 5 days of stimulation with N-CM, L-CM, or H-CM (*n* = 10, 5, and 5, respectively). Red, mitochondria; green, lysosomes; blue, nucleus. **(F–I)** Proteins associated with PINK1/Parkin-mediated mitophagy in the TCs stimulated with the cluster medium only, cluster medium with L-Exo, or cluster medium with H-Exo for 5 days were tested by western blot and quantified using ImageJ (*n* = 3 or 4). **(J)** The colocalization of mitochondria and lysosomes in the TCs stimulated with the cluster medium only, cluster medium with L-Exo, or cluster medium with H-Exo for 5 days was assessed by fluorescence microscopy (*n* = 5, 8, and 8, respectively). Red, mitochondria; green, lysosomes; blue, nucleus. CM, conditioned medium; TCs, target cells; N-CM, normal culture medium; L-CM, conditioned medium from L-SCCs; H-CM, conditioned medium from H-SCCs; L-Exo, exosomes secreted by L-SCCs; H-Exo, exosomes secreted by H-SCCs; the blue triangle represents the expected band for PINK1 or Parkin. Scale bar, 20 μm. *P < 0.05; **P < 0.01; ***P < 0.001.

Next, we investigated whether a supplement with L-CM or H-CM affected PINK1/Parkin-mediated mitophagy in TCs. Western blot results showed that the expression of PINK1, Parkin, and LC3-II was higher in the L-CM- or H-CM-stimulated TCs than in those stimulated with a normal culture medium (N-CM), with H-CM stimulation eliciting the highest levels of expression of these proteins (Figures 3A–D). Fluorescence microscopy also showed a greater level of mitochondria/lysosome colocalization in the TCs stimulated with L-CM or H-CM than in those stimulated with N-CM, with the highest degree of colocalization being observed under H-CM stimulation (Figure 3E). Consistent with the results from the conditioned medium treatment, TCs stimulated with L-Exo or H-Exo also showed higher expression of PINK1, Parkin, and LC3-II than the TCs exposed to an exosome-free medium, with the highest expression levels being seen with H-Exo stimulation (Figures 3F–I). Fluorescence microscopy also demonstrated that both L-Exo and H-Exo stimulation elicited greater levels of mitochondria/lysosome colocalization, with the highest degree of colocalization being recorded with H-Exo stimulation (Figure 3J).

### PINK1/Parkin-Mediated Mitophagy Regulates the Osteogenic Differentiation of Single-Cell Colonies *in vitro*

**FIGURE 4:**
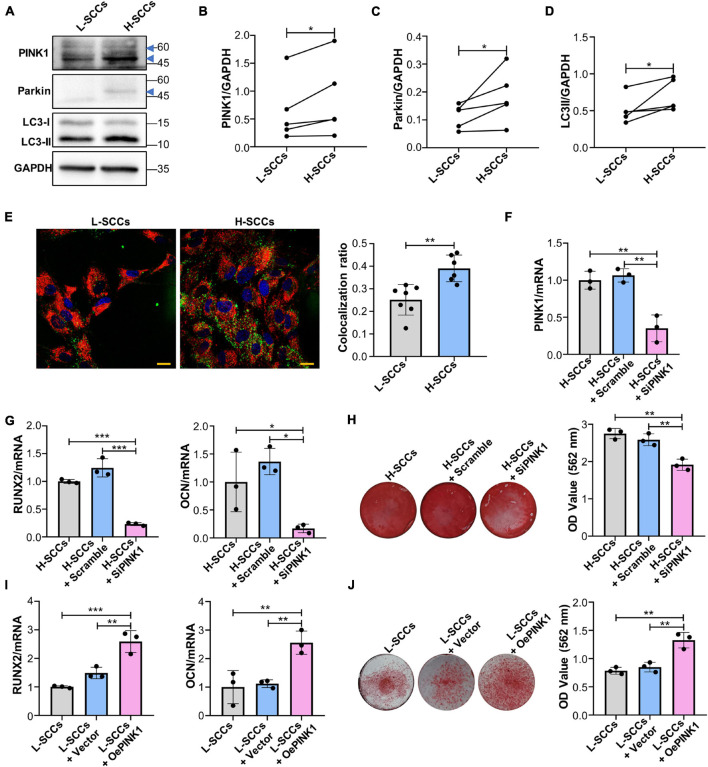
PINK1/Parkin-mediated mitophagy regulated the osteogenic differentiation of SCCs *in vitro*. **(A–D)** Proteins associated with PINK1/Parkin-mediated mitophagy in H-SCCs and L-SCCs were tested by western blot and quantified using ImageJ (*n* = 5). **(E)** The colocalization of mitochondria and lysosomes in H-SCCs and L-SCCs was tested by fluorescence microscopy (*n* = 7 and 6, respectively). Red, mitochondria; green, lysosomes; blue, nucleus. **(F)** PINK1 mRNA expression levels in the H-SCCs or H-SCCs transfected with scrambled siRNA or PINK1 siRNA were measured by RT-qPCR (three independent experiments were performed, with similar results; data are from a single experiment performed in triplicate). **(G)** The expression levels of the osteogenic differentiation-related genes in the H-SCCs or H-SCCs transfected with scrambled siRNA or PINK1 siRNA were measured by RT-qPCR after 7 days of osteogenic induction (three independent experiments were performed, with similar results; data are from a single experiment performed in triplicate). **(H)** The formation of mineralization nodules in the H-SCCs or H-SCCs transfected with scrambled siRNA or PINK1 siRNA was tested by Alizarin red staining after 28 days of osteogenic induction (*n* = 3). **(I)** The expression levels of osteogenic differentiation-related genes in PINK1-overexpressing L-SCCs were determined by RT-qPCR (*n* = 3). **(J)** Mineralized nodule formation in PINK1-overexpressing L-SCCs was tested by Alizarin red staining (*n* = 3). TCs, target cells; L-Exo, exosomes secreted by L-SCCs; H-Exo, exosomes secreted by H-SCCs; H-SCCs + scramble, H-SCCs transfected with scrambled siRNA; H-SCCs + siPINK1, H-SCCs transfected with PINK1 siRNA; L-SCCs + vector, L-SCCs transfected with empty vector; L-SCCs + oePINK1, L-SCCs transfected with a PINK1 overexpression plasmid; the blue triangle represents the expected band for PINK1 or Parkin. Scale bar, 20 μm. *P < 0.05; **P < 0.01; ***P < 0.001.

To determine whether PINK1/Parkin-mediated mitophagy regulates the osteogenic differentiation in SCCs, the levels of PINK1/Parkin-mediated mitophagy in H-SCCs and L-SCCs were assayed by western blot. The results showed that the expression of PINK1, Parkin, and LC3-II was higher in H-SCCs than in L-SCCs (Figures 4A–D). Fluorescence microscopy also showed greater levels of mitochondria/lysosome colocalizations in H-SCCs (Figure 4E). These results indicated that PINK1/Parkin-mediated mitophagy was activated to a greater extent in H-SCCs compared to L-SCCs. Next, we used PINK1-targeting siRNA to inhibit PINK1/Parkin-mediated mitophagy. QPCR analysis confirmed the silencing of PINK1 in H-SCCs (Figure 4F). Osteogenic differentiation analysis demonstrated that the inhibition of PINK1/Parkin-mediated mitophagy not only downregulated *RUNX2* and *OCN* expression (Figure 4G) but also inhibited the formation of mineralized nodules in H-SCCs (Figure 4H). We also overexpressed PINK1 to activate PINK1/Parkin-mediated mitophagy in L-SCCs (Supplementary Figures 3A–E), with the results showing that the activation of PINK1/Parkin-mediated mitophagy led to the upregulation of *RUNX2* and *OCN* expression (Figure 4I) and promoted the formation of mineralized nodules in L-SCCs (Figure 4J). Combined, these results demonstrated that PINK1/Parkin-mediated mitophagy regulates the osteogenic differentiation of SCCs *in vitro*.

### Exosomes Promotes the Osteogenic Differentiation of Target Cells Through PINK1/Parkin-Mediated Mitophagy

**FIGURE 5:**
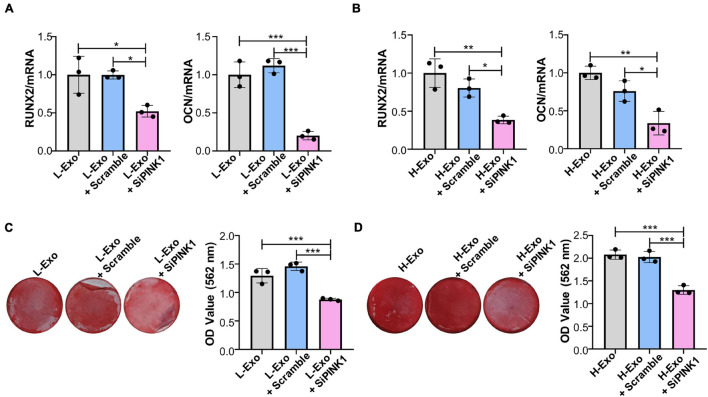
H-Exo and L-Exo promoted the osteogenic differentiation of TCs through PINK1/Parkin-mediated mitophagy. **(A)** Osteogenic inducing fluid containing 20 μg/ml exosomes was used, and the expression levels of the osteogenic differentiation-related genes in the TCs without transfection or transfected with scrambled siRNA or PINK1 siRNA and with L-Exo stimulation were determined by RT-qPCR after 7 days of osteogenic induction (*n* = 3). **(B)** Osteogenic inducing fluid containing 20 μg/ml exosomes was used, and the expression levels of osteogenic differentiation-related genes in the TCs without transfection or transfected with scrambled siRNA or PINK1 siRNA and with H-Exo stimulation were determined by RT-qPCR after 7 days of osteogenic induction (*n* = 3). **(C)** Osteogenic inducing fluid containing 20 μg/ml exosomes was used, and mineralization nodule formation in the TCs without transfection or transfected with scrambled siRNA or PINK1 siRNA and with L-Exo stimulation was tested by Alizarin red staining after 28 days of osteogenic induction (*n* = 3). **(D)** Osteogenic inducing fluid containing 20 μg/ml exosomes was used, and mineralization nodule formation in the TCs without transfection or transfected with scrambled siRNA or PINK1 siRNA and with H-Exo stimulation was tested by Alizarin red staining after 28 days of osteogenic induction (*n* = 3). L-Exo, exosomes secreted by L-SCCs; L-Exo + scramble, TCs transfected scrambled siRNA with L-Exo stimulation; L-Exo + siPINK1, TCs transfected PINK1 siRNA with L-Exo stimulation; H-Exo, exosomes secreted by H-SCCs; H-Exo + scramble, TCs transfected scrambled siRNA with H-Exo stimulation; H-Exo + siPINK1, TCs transfected PINK1 siRNA with H-Exo stimulation. *P < 0.05; **P < 0.01; ***P < 0.001.

To assess whether H-Exo and L-Exo promoted the osteogenic differentiation of TCs through PINK1/Parkin-mediated mitophagy, we knocked down PINK1 in TCs during exosome stimulation. RT-qPCR analysis showed that PINK1 inhibition downregulated the expression of *RUNX2* and *OCN* in both the L-Exo- and H-Exo-stimulated TCs (Figures 5A,B). Alizarin red staining analysis further demonstrated that the silencing of PINK1 could inhibit the L-Exo and H-Exo-stimulated induction of osteogenic differentiation (Figures 5C,D).

### The Activation of PINK1/Parkin-Mediated Mitophagy Promotes Osteogenesis in Single-Cell Colonies With Low Osteogenic Ability *in vivo*

**FIGURE 6:**
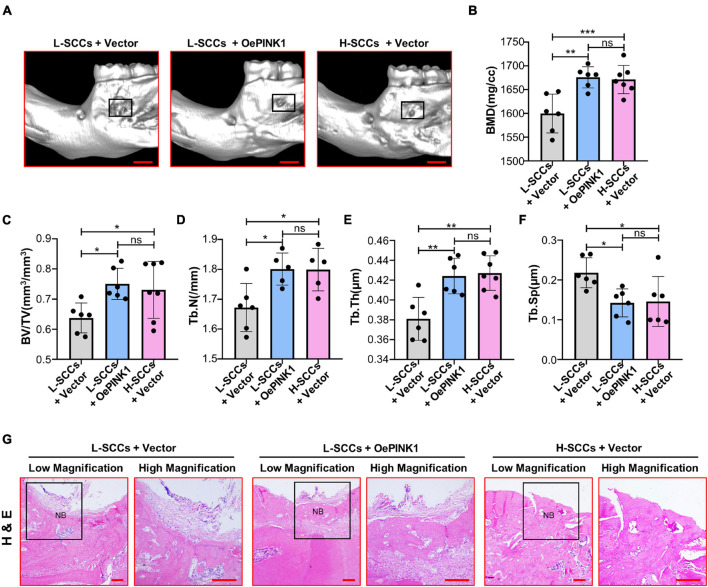
Activation of PINK1/Parkin-mediated mitophagy promoted the osteogenesis of L-SCCs *in vivo*. **(A)** The defects filled with cell sheets derived from empty vector-overexpressing L-SCCs, PINK1-overexpressing L-SCCs, or empty vector-overexpressing H-SCCs were explored by a Micro-CT. **(B–F)** After the defects had been filled in with cell sheets derived from empty vector-overexpressing L-SCCs, PINK1-overexpressing L-SCCs, or empty vector-overexpressing H-SCCs, bone mineral density (BMD), bone volume/total volume (BV/TV), trabecular number (Tb.N), trabecular thickness (Tb.Th), and trabecular separation (Tb.Sp) were evaluated (*n* = 5 to 7). **(G)** Hematoxylin and eosin (H&E) staining was performed 4 weeks after the defects had been filled in with cell sheets derived from empty vector-overexpressing L-SCCs, PINK1-overexpressing L-SCCs, or empty vector-overexpressing H-SCCs. L-SCCs + vector, cell sheet derived from L-SCCs transfected with the empty vector; L-SCCs + oePINK1, cell sheet derived from L-SCCs transfected with the PINK1 overexpression plasmid; H-SCCs + vector, cell sheet derived from H-SCCs transfected with the empty vector; NB, new bone formation. Scale bar, 2 mm for Micro-CT and 100 μm for H&E staining. *P < 0.05; **P < 0.01; ***P < 0.001.

Micro-CT analysis showed that BMD, BV/TV, Tb.N, and Tb.Th values were all higher in the cell sheets from empty vector-overexpressing H-SCCs than in those from empty vector-overexpressing L-SCCs, while empty vector-overexpressing H-SCCs exhibited less trabecular separation (Tb.Sp) (Figures 6A–F). Following the activation of PINK1/Parkin-mediated mitophagy through PINK1 overexpression, BMD, BV/TV, TB.N, and Tb.Th values were significantly higher in the cell sheets from PINK1-overexpressing L-SCCs than in those from empty vector-overexpressing L-SCCs (Figures 6A–E), while PINK1-overexpressing L-SCCs also exhibited less Tb.Sp (Figure 6F). Moreover, H&E staining results showed that both empty vector-overexpressing H-SCCs and PINK1-overexpressing L-SCCs displayed a greater degree of new bone formation than empty vector-overexpressing L-SCCs (Figure 6G).

## Discussion

In our study, we focused on the clonal osteogenic heterogeneity of PDLSCs and explored interclonal communication on the osteogenic differentiation among these heterogeneous osteogenic SCCs, as well as the associated underlying mechanisms. We found that there was heterogeneity in the osteogenic differentiation *in vitro* and osteogenesis *in vivo* within PDLSC-derived SCCs, and revealed that this heterogeneity was regulated by PINK1/Parkin-mediated mitophagy. Additionally, our study confirmed that H-SCCs had a greater osteogenic differentiation-promoting potential than L-SCCs, an effect that was exerted through the exosome-mediated PINK1/Parkin-dependent mitophagy.

Substantial attention has been devoted to both basic and applied research relating to MSCs (Levy et al., 2020). However, a lack of knowledge regarding the interclonal heterogeneity within MSCs is a key obstacle to their therapeutic application and renders the results of MSCs-based clinical trials largely incongruent. Few studies have focused on how these heterogeneous SCCs coordinate and influence their respective functions, which greatly limits the efficacy of MSCs-based therapy. Interclonal heterogeneity includes the morphology as well as the proliferative and differentiation ability, among other factors (Muraglia et al., 2000; Rennerfeldt and Van Vliet., 2016). In our study, we used PDLSC-derived SCCs because of their ease of acquisition and high clone-forming abilities. We focused on the osteogenic heterogeneity not only in terms of osteogenic potential both *in vivo* and *in vitro*, but also to address the conflicting results obtained in PDLSCs-based periodontal regeneration (Chen et al., 2016; Tassi et al., 2017).

Cell-to-cell communication is crucial for intercellular coordination and tissue homeostasis (Zepp and Morrisey, 2019). Exosomes have attracted increasing attention for their abilities to be taken up by cells and deliver substances such as mRNA, miRNA, and proteins, and modulate cell functions (He et al., 2018). Some mRNAs found in exosomes secreted by bone marrow-MSCs can reportedly potentiate tubular cell sensitivity to locally produced insulin-like growth factor-1 (Tomasoni et al., 2013). Here, we selected L-SCCs as the TCs, because, compared with H-SCCs, changes in the osteogenic differentiation abilities would be easier to detect in the former. Our study is the first to show that H-SCCs had a greater osteogenic differentiation-promoting ability than L-SCCs, and that exosomes were involved in such regulation.

Mitochondria control ATP production and redox homeostasis, thereby playing a significant role in the regulation of cellular function (Ma et al., 2020). Under conditions of stress, reactive oxygen species can damage the mitochondrial components, and lead to the accumulation of dysfunctional mitochondria. PINK1/Parkin-mediated mitophagy is a key mechanism for sensing cellular stress and removing damaged mitochondria, which helps maintain cellular homeostasis. PINK1/Parkin-mediated mitophagy has been reported to regulate the osteogenic differentiation of dental pulp stem cells through the BMP/Smad pathway (Pei et al., 2018). One study showed that exosomes could improve the function of recipient cells through the regulation of the mitochondrial function (Zhou et al., 2020), suggesting that an exosome-mediated regulation of mitochondrial function may regulate the osteogenic differentiation among heterogeneous osteogenic SCCs. In our study, the highest levels of PINK1/Parkin-mediated mitophagy were found with H-CM- and H-Exo stimulation. Additionally, through gain- and loss-of-function experiments, including PINK1 knock-down in exosome-stimulated TCs, we demonstrated that PINK1/Parkin-mediated mitophagy regulated the osteogenic differentiation of SCCs.

In summary, our study is the first to show that intercellular communication among heterogeneous PDLSCs is regulated by an exosome-mediated PINK1/Parkin-dependent mitophagy. Our results further indicated that H-SCCs maintain the mitophagy levels in PDLSCs in an exosome-dependent manner, thereby regulating the osteogenic differentiation of PDLSCs.

## Data Availability Statement

The datasets used and/or analyzed during the current study are available from the corresponding author on reasonable request.

## Ethics Statement

The studies involving human participants were reviewed and approved by the Fourth Military Medical University. The patients/participants provided their written informed consent to participate in this study. The animal study was reviewed and approved by the Animal Care Committee of the Fourth Military Medical University.

## Author Contributions

DF contributed to the conception, design, data acquisition, and analysis, and drafted and critically revised the manuscript. YX contributed to the conception, data acquisition, and analysis, and critically revised the manuscript. QZ contributed to the data acquisition and analysis, and critically revised the manuscript. YW, FZ, WZ, and XH contributed to the data acquisition and interpretation, and critically revised the manuscript. QW, YJ, and BL contributed to the conception and design, and drafted and critically revised the manuscript. All authors contributed to the article and approved the submitted version.

## Conflict of Interest

The authors declare that the research was conducted in the absence of any commercial or financial relationships that could be construed as a potential conflict of interest.

## Publisher’s Note

All claims expressed in this article are solely those of the authors and do not necessarily represent those of their affiliated organizations, or those of the publisher, the editors and the reviewers. Any product that may be evaluated in this article, or claim that may be made by its manufacturer, is not guaranteed or endorsed by the publisher.
