# Supplementary material for: Exosomes Regulate Interclonal Communication on Osteogenic Differentiation Among Heterogeneous Osteogenic Single-Cell Clones Through PINK1/Parkin-Mediated Mitophagy
Source: Front Cell Dev Biol. 2021 Sep 17;9:687258. doi: 10.3389/fcell.2021.687258 (PMC8484762; doi:10.3389/fcell.2021.687258)
Supplement: Supplementary file 1 [file Data_Sheet_1.pdf]

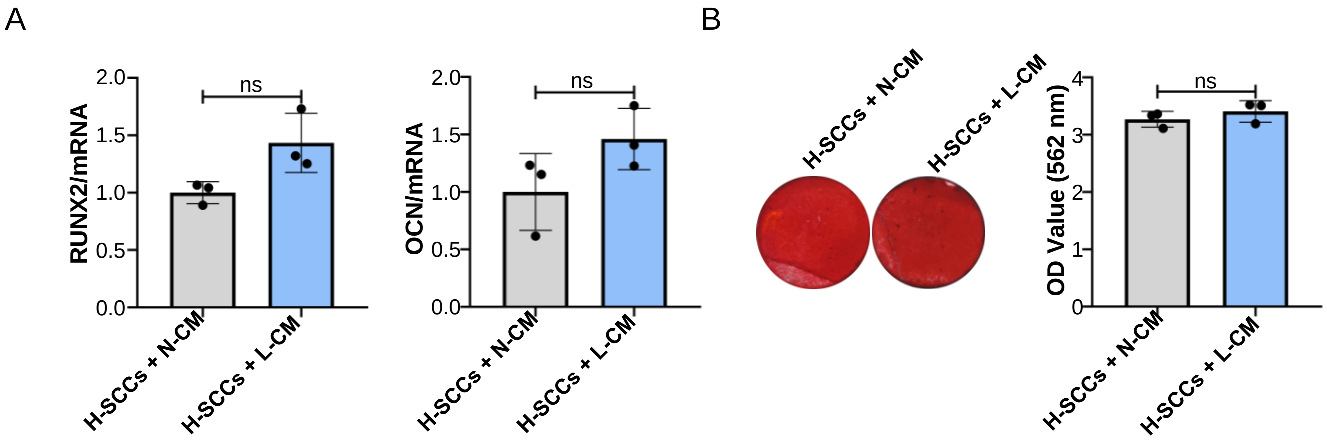

Figure S1. The effect of L-CM on the osteogenic differentiation of H-SCCs. (A) RT-qPCR was performed to investigate the expressions of *RUNX2* and *OCN* in H-SCCs stimulated with two-fold concentrated osteogenic induction medium mixed with equal volumes of N-CM or L-CM (*n*=3). (B) Mineralized nodule formation in H-SCCs stimulated with two-fold concentrated osteogenic induction medium mixed with equal volumes of N-CM or L-CM was tested by Alizarin red staining (*n*=3). N-CM: normal culture medium; L-CM: conditioned medium from L-SCCs.

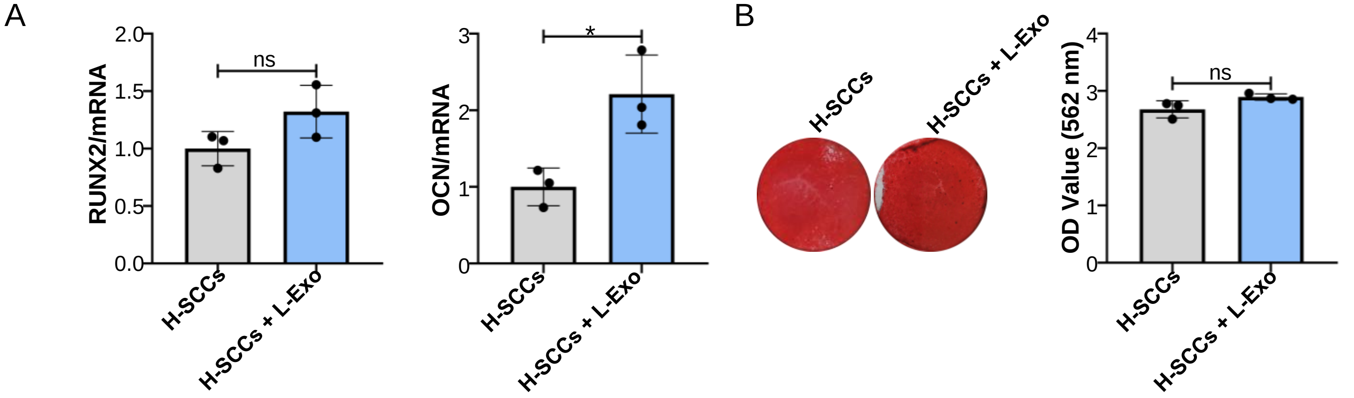

Figure S2. The effect of L-Exo on the osteogenic differentiation of H-SCCs. (A) RT-qPCR was performed to investigate the expressions of *RUNX2* and *OCN* in H-SCCs stimulated by osteogenic inducing fluid without exosomes or osteogenic inducing fluid containing 20 μg/mL L-Exo (*n*=3). (B) Mineralized nodule formation in H-SCCs stimulated by osteogenic inducing fluid without exosomes or osteogenic inducing fluid containing 20 μg/mL L-Exo was tested by Alizarin red staining (*n*=3). L-Exo: exosomes secreted by L-SCCs.

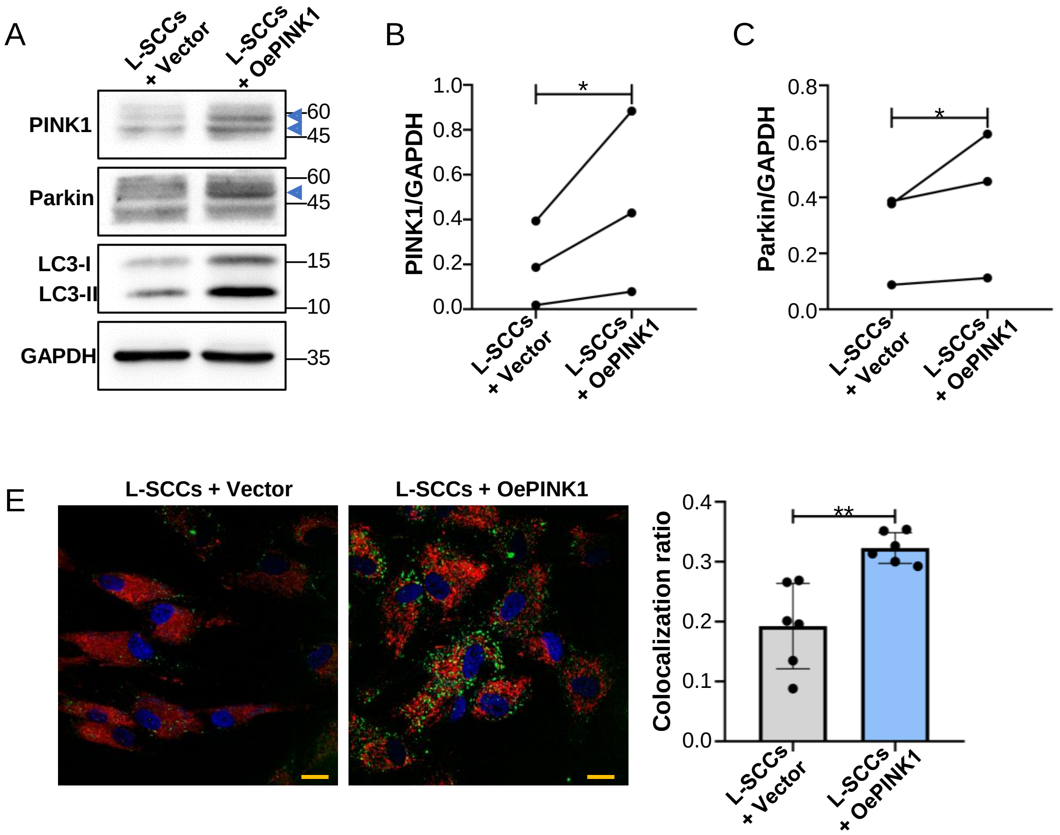

Figure S3. Overexpression of PINK1 leads to activation of PINK1/Parkin-mediated mitophagy in L-SCCs. (A-D) Proteins associated with PINK1/Parkin-mediated mitophagy in PINK1-overexpressing L-SCCs were tested by Western blot and quantified using ImageJ (*n*=3). (E) The colocalization of mitochondria and lysosomes was assessed by fluorescence microscopy in PINK1-overexpressing L-SCCs (*n*=6). Red: mitochondria; Green: lysosomes; Blue: nucleus. L-SCCs + vector: L-SCCs transfected with empty vector; L-SCCs + oePINK1: L-SCCs transfected with a PINK1 overexpression plasmid; the blue triangle represents the expected band for PINK1 or Parkin; scale bar: 20 μm.
